# Circularly polarized luminescent systems fabricated by Tröger's base derivatives through two different strategies

**DOI:** 10.3762/bjoc.17.6

**Published:** 2021-01-06

**Authors:** Cheng Qian, Yuan Chen, Qian Zhao, Ming Cheng, Chen Lin, Juli Jiang, Leyong Wang

**Affiliations:** 1 Key Laboratory of Mesoscopic Chemistry of MOE, Jiangsu Key Laboratory of Advanced Organic Materials, School of Chemistry and Chemical Engineering, Nanjing University, Nanjing, 210023, China; 2 Advanced Materials Institute, Qilu University of Technology (Shandong Academy of Sciences), Jinan, 250014, China

**Keywords:** circularly polarized luminescence, chiral resolution, co-gelation, inversion of CPL handedness, Tröger's base

## Abstract

The Tröger's base derivative *rac*-**TBPP** was synthesized and separated into two enantiomers *R_2N_*-**TBPP** and *S_2N_*-**TBPP** by chiral column chromatography. These compounds show a strong circularly polarized luminescence with *g*_lum_ values of +0.0021, and −0.0025, respectively. The second way to fabricate the *rac*-**TBPP**-based CPL-active material is to co-gel the fluorescent *rac*-**TBPP** with a chiral ᴅ-glutamic acid gelator **DGG** by co-assembly strategy. At the molar ratio of *rac*-**TBPP**/**DGG** = 1:80, the *g*_lum_ value of the co-gel was about three times higher than the *g*_lum_ values of *R_2N_*-**TBPP** and *S_2N_*-**TBPP** enantiomers. Interestingly, the CPL handedness of the *rac*-**TBPP**/**DGG** co-gel could be adjusted effectively by changing their stoichiometric ratios.

## Introduction

Recently, much effort has been devoted to constructing luminescent materials with efficient high emission in the solid state [1–3]. More and more types of fluorophores with aggregation-induced emission (AIE) characteristics have been discovered and applied in practice [4–6]. Among them, the fluorescent materials emitting circularly polarized luminescence (CPL) have attracted intensive interest owing to their wide applications in various researching fields including 3D displays, chiroptical materials, and so on [7–10]. Circular dichroism (CD) absorption spectra reflect the chirality of the fluorescent materials in the ground state, and circularly polarized luminescence (CPL) spectra reflect the chirality of fluorescent materials in the excited electronic state. Therefore, the CD and CPL spectrum are the two most important tools to test the chirality of luminescent materials [11–12].

**Scheme 1:**
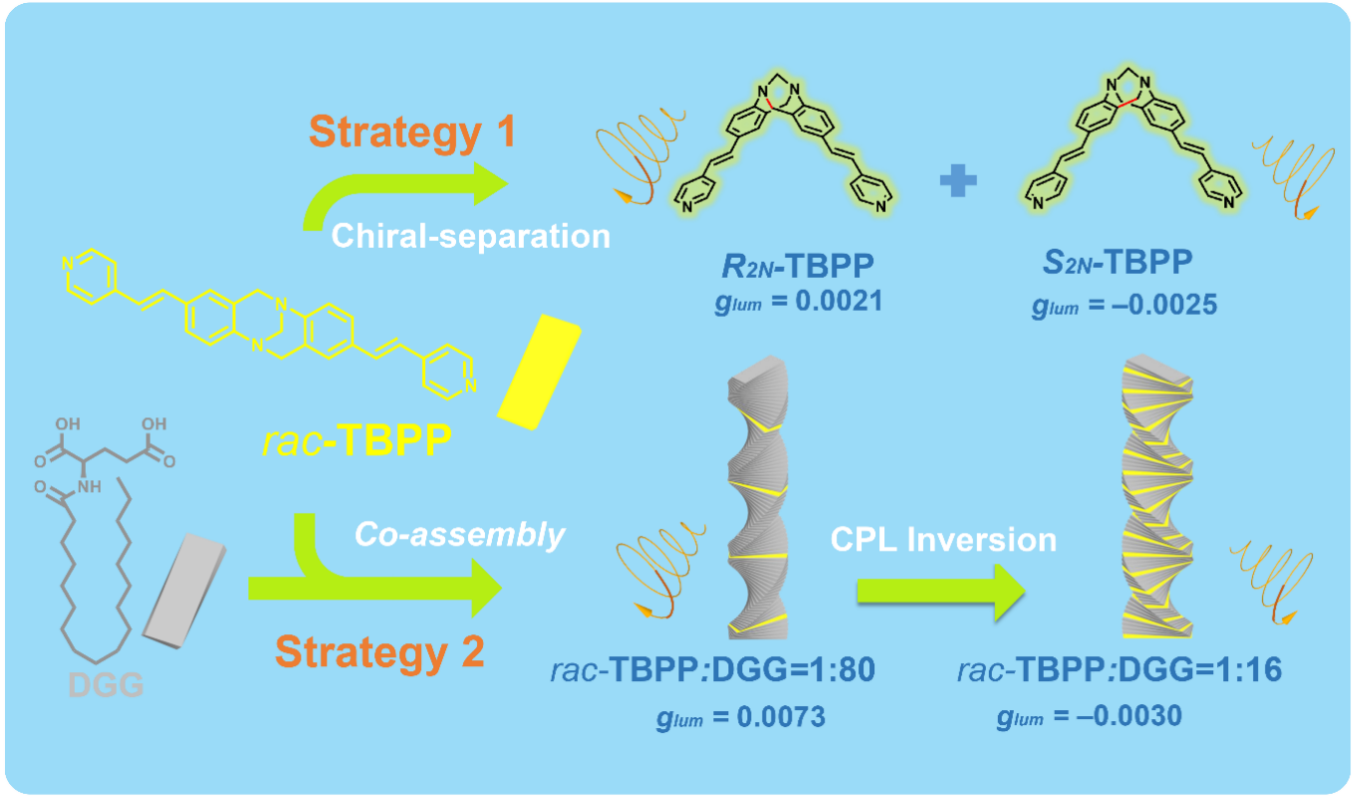
Cartoon representative for *rac*-**TBPP**-based CPL-active systems fabricated through two strategies.

As a useful building block in constructing functional materials [13–14], Tröger’s base (TB), first synthesized in 1887 [15], shows high controllability and obvious advantages. There are eleven sites in its framework that could be modified without considering the side chain. At the same time, the loose stacking of the TB unit and its derivatives, which is caused by its V-configuration could reduce the distance-dependent intermolecular quenching effect in the aggregation state [16]. Moreover, the large dihedral angle of TB (80–104°) [15] permits less self-absorption and a wider stokes shift [17]. Further, the steric hindrance and highly rigidity could reduce non-radiative transition and restrict the internal rotation [18]. More importantly, in the TB structure, its bridged methylene groups of the diazocine chiral nitrogen atoms prevent the inversion of the configuration, and two stable enantiomers could be formed and separated then. Although the TB shows excellent performance in constructing AIE materials, TB-based materials emitting CPL have rarely been reported so far. In general, the luminescent part and the chiral part are necessary to construct CPL-active materials [19–21], so the fluorescent Tröger's base derivatives *rac-***TBPP** fall into our sight as the candidate to construct CPL-active materials. Herein, we take two stratgies to construct *rac-***TBPP**-based CPL material. One stratgy is to separate non-CPL emission *rac-***TBPP** into CPL-active enantiomers *R_2N_-***TBPP** and *S_2N_-***TBPP**, respectively. The other stratgy is to co-assemble the fluorescent *rac-***TBPP** with the chiral ᴅ-glutamic acid gelator **DGG** to form the CPL-active co-gel. Interestingly, adjusting the stoichiometric ratios of *rac*-**TBPP**/**DGG** of the co-assembling system, the handedness of CPL-active co-gel can be controlled effectively (Scheme 1).

## Results and Discussion

**Figure 1:**
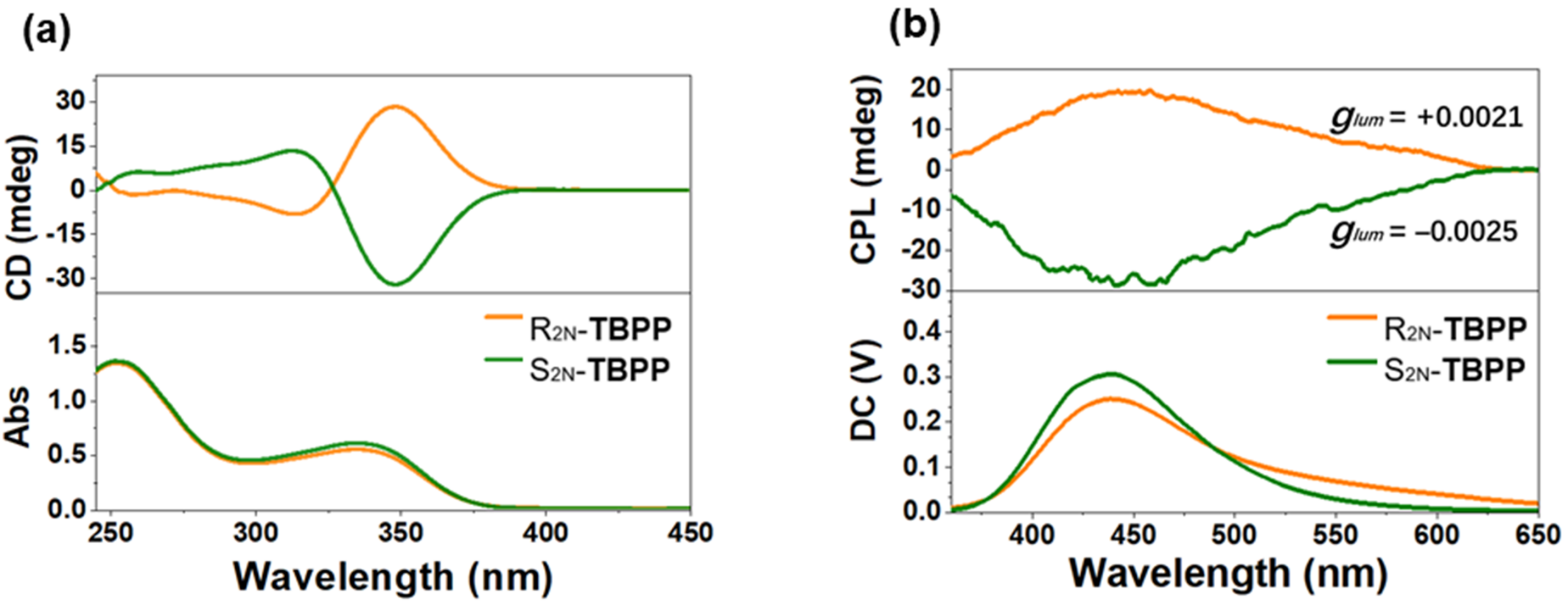
Ground-state and excited-state chirality of *R_2N_-***TBPP** and *S_2N_-***TBPP**. (a) CD spectra of *R_2N_-***TBPP** and *S_2N_-***TBPP**. (b) CPL spectra of *R_2N_-***TBPP** and *S_2N_-***TBPP** excited at 350 nm.

The synthetic routes of *rac-***TBPP** are outlined in Supporting Information File 1, Scheme S1. Firstly, 2,8-dibromo-6*H*,12*H*-5,11-methanodibenzo[*b*,*f*][1,5]diazocine was synthesized according to the reported procedure [22]. Then, by Suzuki coupling reaction between 2,8-dibromo-6*H*,12*H*-5,11-methanodibenzo[*b*,*f*][1,5]diazocine and 4-vinylpyridine, *rac-***TBPP** was successfully obtained in 51.8% yield. Detailed experiments and characterization were described in Supporting Information File 1 (Figures S1–S3). Firstly, *rac-***TBPP** was separated into two fractions *R_2N_-***TBPP** and *S_2N_-***TBPP** by a chiralpak IB column using MeOH/DCM (80:20, v/v) as the eluent (Supporting Information File 1, Figure S5). The CD spectrum of the first fraction exhibited a positive Cotton effect at 352 nm, assigned to *R_2N_-***TBPP**, while the second one showed a negative Cotton effect at the same wavelength, assigned to *S_2N_-***TBPP** (Figure 1a) [23]. *R_2N_-***TBPP** and *S_2N_-***TBPP** were tested further by CPL spectroscopy, and the magnitude of the CPL emission was estimated by a luminescence dissymmetry factor (*g*_lum_), defined as 2(*I*_L_ − *I*_R_ )/(*I*_L_ + *I*_R_) where *I*_L_ and *I*_R_ are the intensity of the left-handed and right-handed CPL signals [24], respectively. Ranging from +2 for an ideal left-handed CPL to −2 for an ideal right-handed CPL, the *g*_lum_ value comes up to zero when no circular polarization of the luminescence was detected. The calculated value of *g*_lum_ of the CPL signals for *R_2N_-***TBPP** and *S_2N_-***TBPP** are +0.0021, and −0.0025, repectively (Figure 1b), which is larger than many small organic molecules [25].

**Figure 2:**
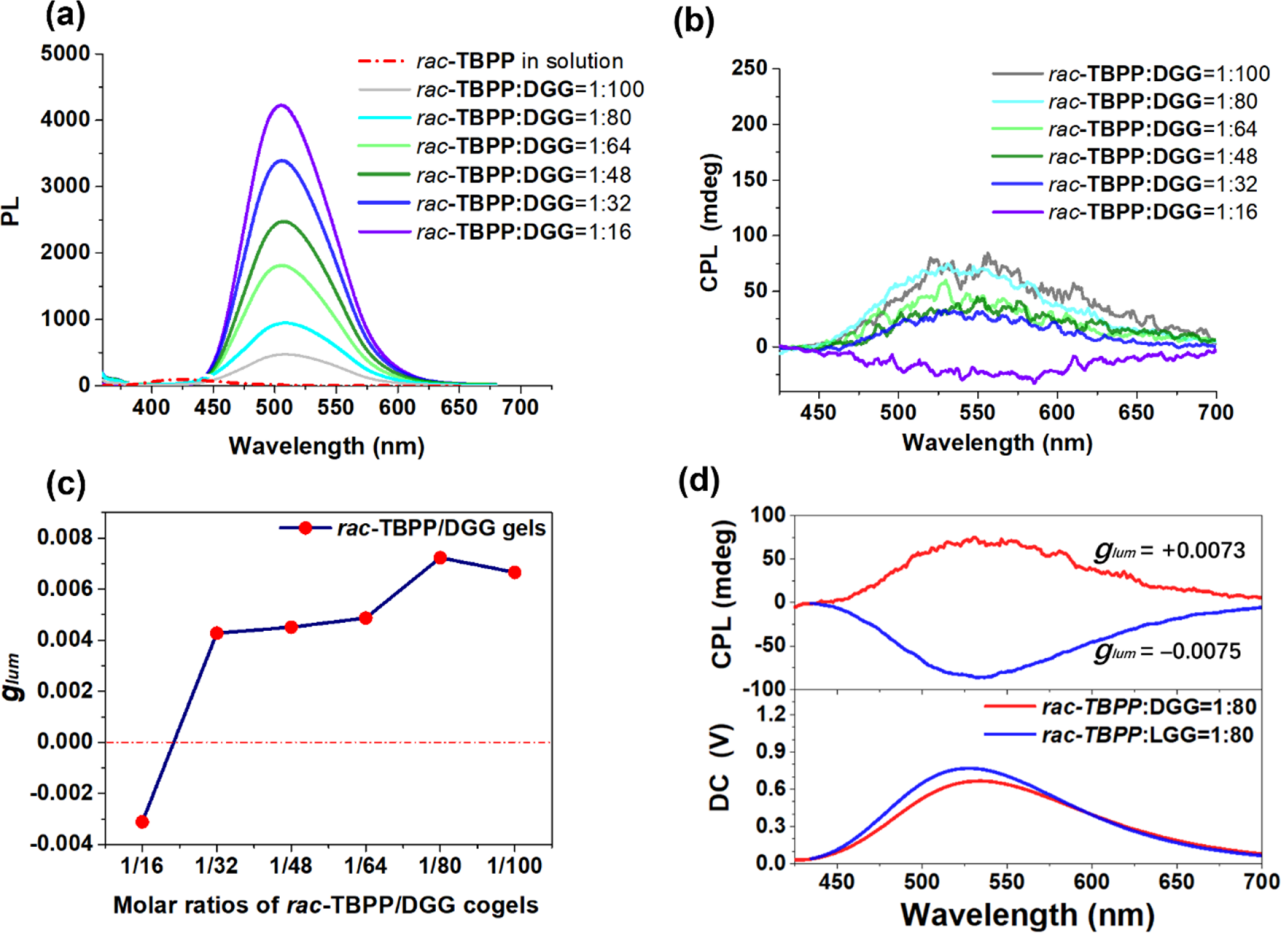
(a) Fluorescence spectra of *rac*-**TBPP** in solution (dash line) and in the *rac*-**TBPP**/**DGG** co-gels. (b) CPL spectra of the *rac*-**TBPP**/**DGG** co-gel at molar ratios from 1:100 to 1:16. (c) Plot of *g*_lum_ value of CPL signals versus ratios of *rac*-**TBPP**/**DGG** in the co-gel. (d) Mirror symmetry in CPL spectra of *rac*-**TBPP**/**DGG** co-gel (red line) and *rac*-**TBPP**/**LGG** (blue line) co-gel at the molar ratio of 1:80.

In order to avoid a tedious chiral separation of *rac-***TBPP**, we tried to construct the CPL-active material by co-assembling the achiral fluorophore *rac-***TBPP** with a chiral gelator **DGG**. In the CPL-active co-gels, noncovalent weak interactions might be formed between achiral fluorophores and a chiral gelator. So, the CPL emission of co-gels could be adjusted easily by external stimuli. The ᴅ-glutamic acid gelator **DGG** and its enantiomer **LGG** possess three hydrogen-bond sites, two carboxylic acid groups and one amide, which could be assemblied into the stable spiral structure by hydrogen-bond and other noncovlant interactions. **DGG** was synthesised by introducing an octadecyl moiety into the glutamic skeleton in 78.6% yield according to the reported route (Supporting Information File 1, Scheme S2) [26]. When *rac-***TBPP** was mixed with **DGG** at molar ratios from 1:100 to 1:16 (*rac-***TBPP**/**DGG**), transparent yellow co-gels were successfully formed by being heated to dissolve in chloroform, and then cooled to ambient temperature (Supporting Information File 1, Figure S6). Owing to the AIE effect of the TB unit the fluorescence intensity of these co-assembly co-gels enhanced sharply (Figure 2a). More interestingly, at the molar ratio of *rac*-**TBPP**/**DGG** = 1:16, the CPL spectra of the co-gel shows a negative signal, while at the molar ratio of *rac*-**TBPP**/**DGG** = 1:32 or higher, positive signals exhibiting left-handed CPL signals were observed (Figure 2b). At the molar ratio of *rac*-**TBPP**/**DGG** = 1:80, the CPL spectra shows a positive signal with a *g*_lum_ value about +0.0073, which was almost three times higher than the *g*_lum_ value of **TBPP** enantiomers (Figure 2c). Compared with the co-gel at the molar ratio of *rac*-**TBPP**/**DGG** = 1:80, a mirror symmetry was observed in the CPL spectra of the co-gel at the molar ratio of *rac*-**TBPP**/**LGG** = 1:80 (Figure 2d).

**Figure 3:**
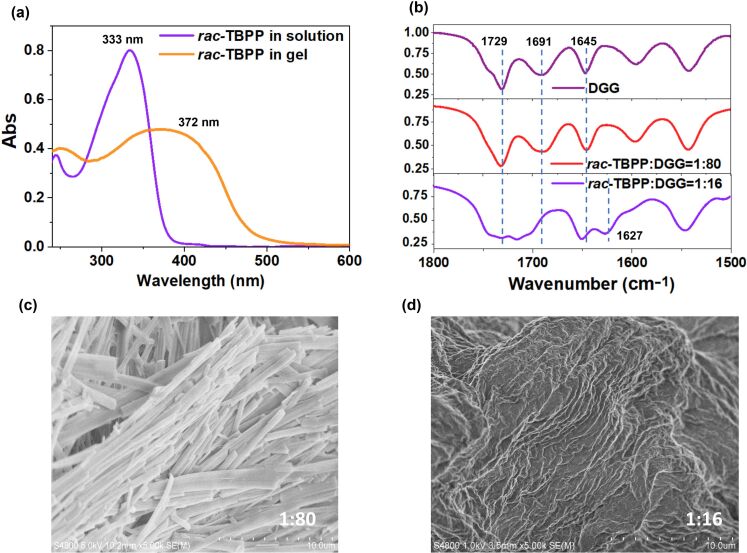
(a) UV–vis absorption spectra of *rac-***TBPP** and *rac-***TBPP**/**DGG** co-gel (*rac-***TBPP**/**DGG** = 1:80). (b) FTIR spectra of **DGG** and *rac-***TBPP**/**DGG** co-gels at molar ratios of 1:80 and 1:16, respectively. SEM images of *rac-***TBPP**/**DGG** co-gels at molar ratios of 1:80 (c) and 1:16 (d).

*rac-***TBPP** contains pyridine units, and the gelator **DGG** has the carboxyl groups. Therefore, hydrogen bonds might be formed between pyridine in *rac-***TBPP** and the carboxyl groups in **DGG**. Moreover, the morphologies of *rac-***TBPP**/**DGG** co-gels might be different due to the changing stoichiometric ratios. In order to get an indepth understanding on the inversion of CPL handedness, *rac-***TBPP**/**DGG** co-gels at molar ratios of 1:16 and 1:80 were explored further by UV–vis and FTIR (fourier transform infrared) spectra. UV–vis absorption spectra of *rac-***TBPP**/**DGG** co-gels exhibited a strong absorption band at 333 nm, assigned to the conjugated structure of benzene and pyridine in the *rac-***TBPP** (Figure 3a). However, a red-shift broaden absorption band situated at 372 nm appears in the *rac-***TBPP**/**DGG** co-gel, implying the formation of the ordered packing of *rac-***TBPP** in supramolecular assemblies. At the molar ratio of *rac-***TBPP**/**DGG** = 1:80, The FTIR spectrum was similar to that of the **DGG** gel, in which ν*_C=O_* bonds at 1729, 1691, and 1645 cm^−1^ reveal that the carboxyl acid groups of **DGG** could be involved in the formation of various hydrogen bonds (Figure 3b, Figure S7, Supporting Information File 1). At the molar ratio of *rac-***TBPP**/**DGG** = 1:16, the intensity of the peak at 1691 cm^−1^ decreases, and the peak at 1729 cm^−1^ brodens. A new peak adjacent to 1645 cm^−1^ appears at 1627 cm^−1^. The results demonstrate that some of the acid–acid hydrogen bonds between **DGG** molecules might be replaced by acid–pyridine hydrogen bonds between DGG and *rac-***TBPP** [27]. In addition, the possible influence of the stoichiometric ratios to the morphologies of *rac-***TBPP/DGG** co-gels was investigated using a scanning electron microscope (SEM). At the molar ratio of *rac-***TBPP**/**DGG** = 1:80, the co-gel shows belt-like nanofibers (Figure 3c), while the fibrous morphology could not be observed at the molar ratio of 1:16 (Figure 3d). It indicates that two different kinds of supramolecular assemblies were formed at the ratios of *rac-***TBPP**/**DGG** 1:80 and 1:16, respectively, which is also coincident with the inversion of the CPL responses. However, more detailed studies are still needed to clarify the relations between the morphology and CPL handedness.

## Conclusion

In conclusion, two strategies were demonstrated to obtain CPL-active materials based on the Tröger’s base derivative *rac*-**TBPP**. One method is to separate *rac*-**TBPP** into two enantiomers *R_2N_*-**TBPP** and *S_2N_*-**TBPP**, which emit strong circularly polarized luminescence. The other strategy is to co-gel the fluorescent *rac*-**TBPP** with a chiral ᴅ-glutamic acid gelator **DGG** by the co-assembly strategy. The cogels show significant CPL emission and stoichiometry-controlled inversion of chirality due to the hydrogen bonding interactions and packing modes in the supramolecular co-assemblies. Owing to TB special V-shaped structure, rigid conformation, and nitrogen stereogenic centers make it and its derivatives useful building blocks to construct CPL-active materials and to develop chiral phosphorescent materials in future.

## Supporting Information

File 1 Experimental part.
