# Supplementary material for: Circularly polarized luminescent systems fabricated by Tröger's base derivatives through two different strategies
Source: Beilstein J Org Chem. 2021 Jan 6;17:52–7. doi: 10.3762/bjoc.17.6 (PMC7801797; doi:10.3762/bjoc.17.6)
Supplement: File 1 — Experimental part. [file Beilstein_J_Org_Chem-17-52-s001.pdf]

BEILSTEIN JOURNAL OF ORGANIC CHEMISTRY

# Supporting Information

for

# Circularly polarized luminescent systems fabricated by Tröger's base derivatives through two different strategies

Cheng Qian, Yuan Chen, Qian Zhao, Ming Cheng, Chen Lin, Juli Jiang and Leyong Wang

*Beilstein J. Org. Chem.* **2021,** *17,* 52–57. doi:10.3762/bjoc.17.6

# Experimental part

License and Terms: This is a supporting information file under the terms of the Creative Commons Attribution License (https://creativecommons.org/licenses/by/4.0). Please note that the reuse, redistribution and reproduction in particular requires that the author(s) and source are credited and that individual graphics may be subject to special legal provisions.
The license is subject to the *Beilstein Journal of Organic Chemistry* terms and conditions: (https://www.beilstein-journals.org/bjoc/terms)

**Table of Contents**

1. General information ....................S3

2. Synthesis of *rac*-**TBPP** and the gelator **DGG** ....................S3

3. Resolution of the enantiomers *rac*-**TBPP** by chiral HPLC ....................S6

4. Fabrication of the cogels ....................S7

5. FT-IR absorption spectra of *rac*-**TBPP**/**DGG** cogels ....................S8

6. Reference ....................S8

## 1. General information

All reactions were performed in air atmosphere unless otherwise stated. All reagents and solvents, unless otherwise indicated, were obtained from commercial sources. Melting points (M.p.) were determined using a Focus X-4 apparatus (made in China) and were not corrected. All yields were given as isolated yields. NMR spectra were recorded on a Bruker DPX 400 MHz spectrometer tetramethylsilane (TMS) solvent signals as internal references at 298 K. LCMS equipped with an electrospray ionization (ESI) probe operate in positive-ion mode with direct infusion. Fourier transform infrared (FTIR) spectra were recorded using a Thermo Nicolet Nexus FTIR devise with the Smart Golden Gate ATR attachment in the range of 4000–400 $cm^{-1}$ with 2 $cm^{-1}$ resolution. UV–vis and CD spectra were obtained using Hitachi U-3600 spectrophotometer and JASCO J-810 spectrophotometer, respectively. Fluorescence spectra of both the solution and cogels were measured on a F-7000 FL spectrophotometer (HITACHI, Japan) using a xenon lamp as the excitation source. CPL spectra were measured on JASCO CPL-300.

## 2. Synthesis of *rac*-TBPP and the gelator DGG

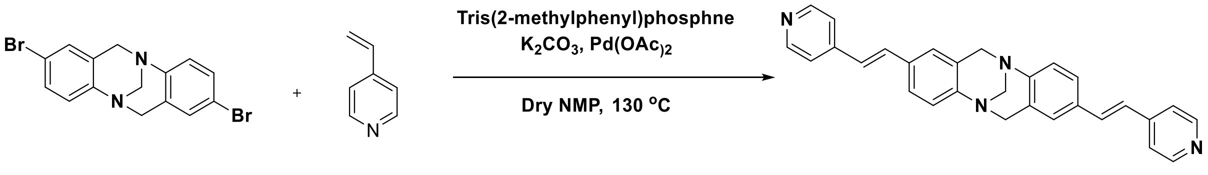


**Scheme S1.**The synthesis procedures of ***rac*-TBPP**.

**Synthesis of *rac*-TBPP and characterization data**

2,8-Dibromo-6*H*,12*H*-5,11-methanodibenzo[*b*,*f*][1,5]diazocine was initially synthesized according to the reported procedures [S1]. *rac*-**TBPP** was synthesized according to the reported literature [S2]. A single-necked flask equipped with a magnetic stirrer was charged with 2,8-dibromo-6*H*,12*H*-5,11-methanodibenzo[*b*,*f*][1,5]diazocine (0.667 mmol, 0.252 g), $K_2CO_3$ (3.76 mmol, 0.52 g), tris(2-methylphenyl)phosphine (1.0 mmol, 0.30 mg) and palladium acetate (1.0 mmol, 2.25 mg) in dry *N*-methylpyrrolidone solution (10 mL) under an $N_2$ atmosphere. Then 4-vinyl pyridine (2.13 mmol, 0.22ml) was added slowly by using a syringe, and the solution was refluxed at 130 °C for 12 h. After the reaction was finished, the reaction mixture was cooled to ambient temperature and extracted by $CH_2Cl_2$ (3 × 100 mL). The organic phase was washed with saturated sodium chloride solution and dried with anhydrous $Na_2SO_4$. After being filtered, the organic solvent was removed using a rotary evaporator, the product was purified by column chromatography on silica gel (eluent: 1:40, *v/v*,

methanol/dichloromethane gradually changing to 1:25) to afford *rac*-**TBPP** as a yellow solid (0.147 g, 0.345 mmol, 51.8 %). M. p.: 286-288 °C. $^{1}H$ NMR (400 MHz, $CDCl_3$) $\delta$ = 8.54 (d, $J$ = 6.0 Hz, 4H), 7.38 (dd, $J_1$ = 8.4 Hz, $J_2$ = 2.0 Hz, 2H), 7.34-7.32 (m, 4H), 7.21-7.10 (m, 6H), 6.87 (d, $J$ = 16.4 Hz, 2H), 4.75 (d, $J$ = 16.4 Hz, 2H), 4.35 (s, 2H), 4.24 (d, $J$ = 16.8 Hz, 2H) ppm. $^{13}C$ NMR (100 MHz, $CDCl_3$): 149.1, 148.6, 146.2, 133.8, 131.8, 128.2, 126.3, 126.0, 125.5, 124.5, 121.0, 66.9, 58.7 ppm. (ESI-MS) m/z: calcd. for $[C_{29}H_{24}N_4 + H]^+$ = 429.20; found: 429.05.

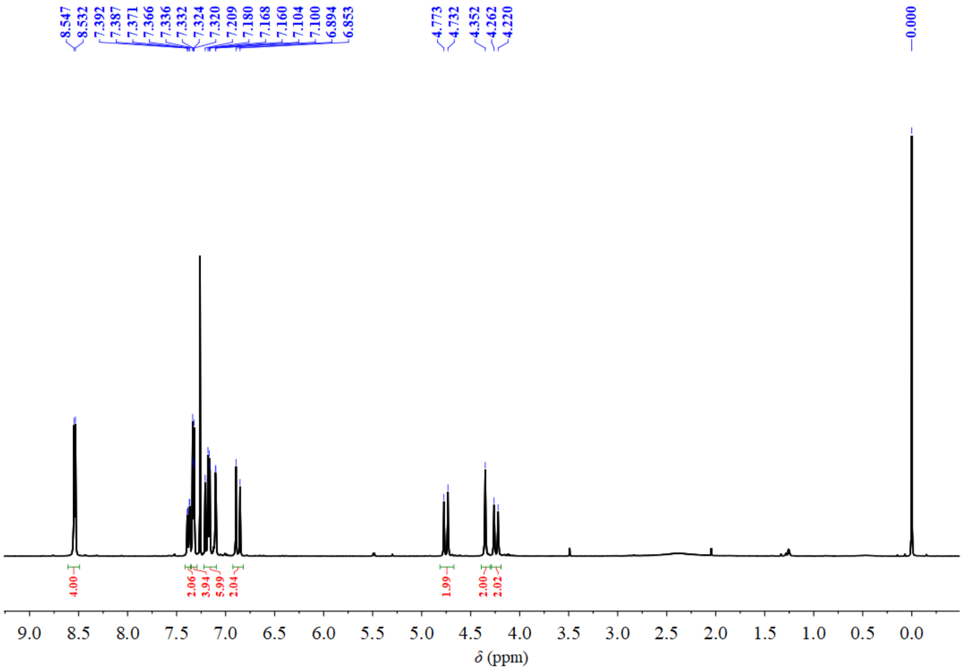

**Figure S1**. $^{1}H$ NMR spectrum (400 MHz, $CDCl_3$, 298 K) of *rac*-**TBPP**.

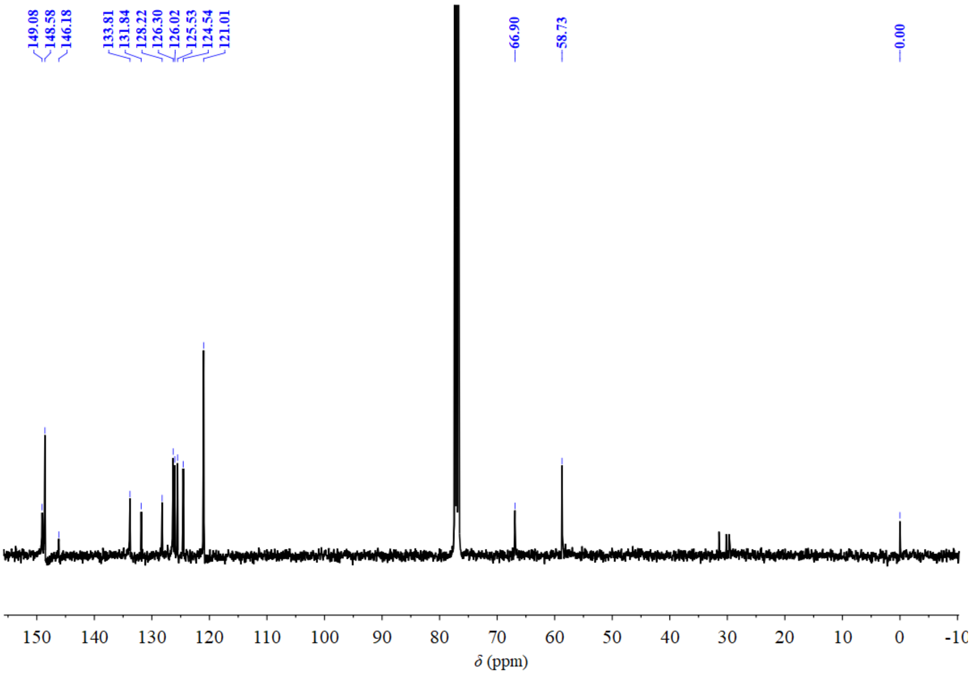

**Figure S2**. $^{13}C$ NMR spectrum (100 MHz, $CDCl_3$, 298 K) of *rac*-**TBPP**.

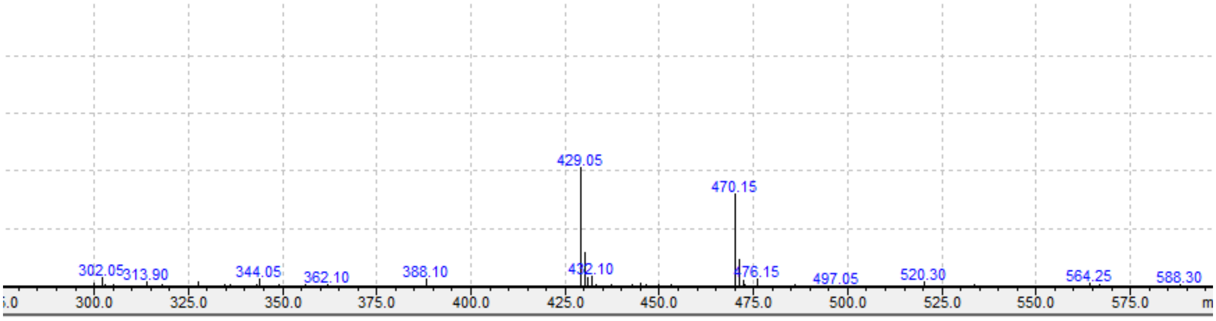


**Figure S3**. ESI-MS of ***rac*-TBPP** with the parent ion $[M+H]^+$ at 429.05

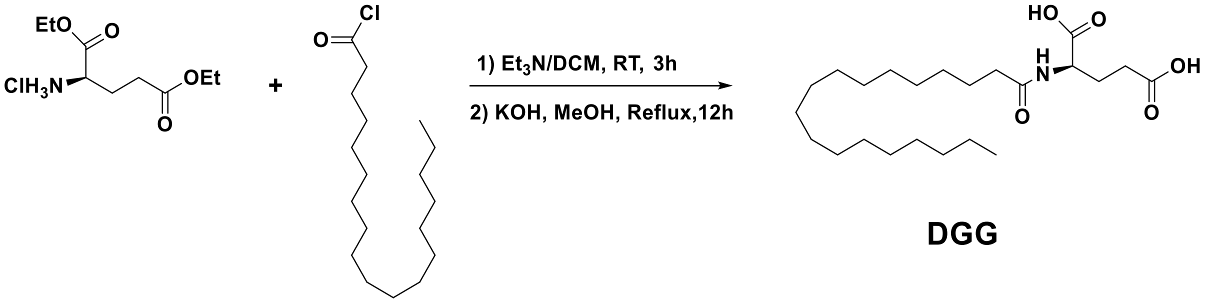


**Scheme S2.** The synthesis procedures of **DGG**.

**Synthesis of the gelator DGG/LGG and characterization data**

**DGG** was prepared according to the previously reported methods [S3].

The compound L-glutamic acid diethyl ester hydrochloride (1.02 g, 4.26 mmol) was dissolved in dry $CH_2Cl_2$ (80 mL) containing $Et_3N$ (1.24 g, 1.61 mL, 12.25 mmol) at 0 °C, and a solution of stearoyl chloride (1.38 ml, 5.01 mmol) in dry $CH_2Cl_2$ (20 mL) was added to the mixture slowly over 1 h. After warming to ambient temperature, the mixed solution was stirred for additional 3 h. Then the reaction was quenched by addition of deionized water (100 mL), and extracted by $CH_2Cl_2$ (2 × 100 mL), the organic phase was dried with anhydrous $Na_2SO_4$. The crude product was obtained after filtration and rotary evaporation. The product was used for next step without purification, which was dissolved in a 1:1 mixture solution of MeOH/water (*v/v*, 80 mL) and KOH (0.32 g, 5.70 mmol) was added. The mixture was heated under reflux for 12 h. After cooling to ambient temperature, MeOH was removed using rotary evaporator, and the aqueous phase was acidified with 2 M HCl to pH = 2.0. The formed precipitate was filtered off, washed with deionized water thoroughly, dried and recrystallized using acetone as solvent to obtain the white solid (1.38 g, 3.34 mmol, 78.6 %). $^1H$ NMR (400 MHz, DMSO-$d_6$) $\delta$ = 12.32 (s, 2H), 8.04 (d, $J$ = 8.0 Hz, 1H), 4.21-4.16 (m, 1H), 2.28-2.24 (m, 2H), 2.12-2.08 (m, 2H), 1.99-1.90 (m, 1H), 1.79-1.70 (m, 1H), 1.49-1.46 (m, 1H), 1.23 (s, 28H), 0.85 (t, $J$ = 6.4 Hz, 3H) ppm. **LGG** was obtained by the same synthetic procedure as above-mentioned for **DGG**.

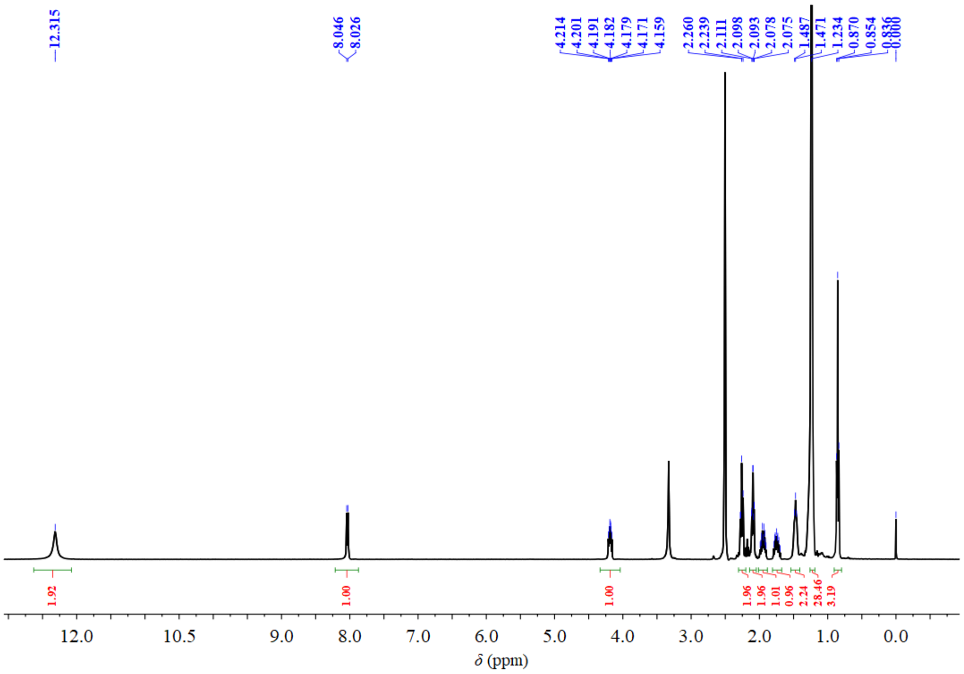

**Figure S4.** $^{1}$H NMR spectrum (400 MHz, DMSO-$d_6$, 298 K) of **DGG**.

## 3. Chiral resolution of *rac*-TBPP by chiral HPLC.

### Optical isomer resolution conditions:

| | | |
|---|---|---|
| Column | : | CHIRALPAK IB N-5(IBN5CD-VD005) |
| Column size | : | 0.46 cm I.D. × 15 cm L |
| Injection | : | 20.0 ul |
| Mobile phase | : | MeOH/DCM=80/20(V/V) |
| Flow rate | : | 1.0 ml/min |
| Wave length | : | UV 25 nm |
| Temperature | : | 35 °C |
| HPLC equipment | : | Shimadzu LC-20AD CP-HPLC-06 |
| Sample name | : | Raw Material |

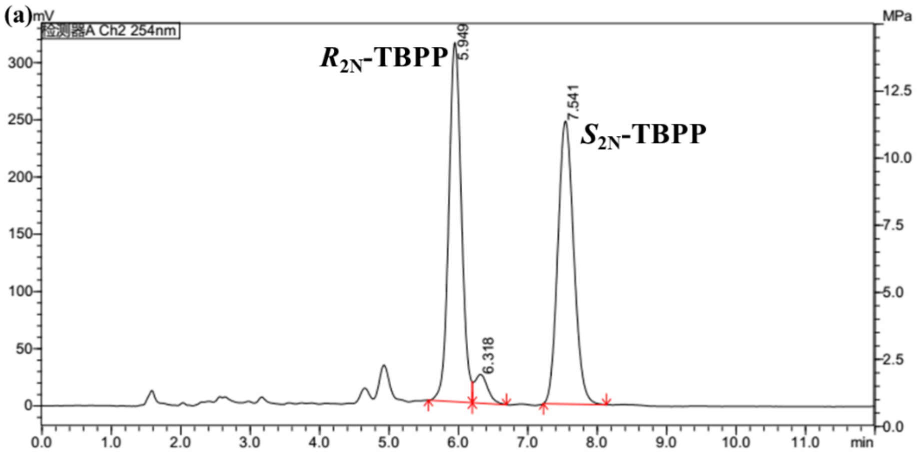


| Peak# | Ret. Time | Area | Area% | T.Plate# | Tailing F. | Resolution |
|---|---|---|---|---|---|---|
| 1 | 5.949 | 3970818 | 48.181 | 5031 | -- | -- |
| 2 | 6.318 | 331918 | 4.027 | 2794 | -- | 0.907 |
| 3 | 7.541 | 3938691 | 47.791 | 5083 | 1.117 | 2.715 |

(b)

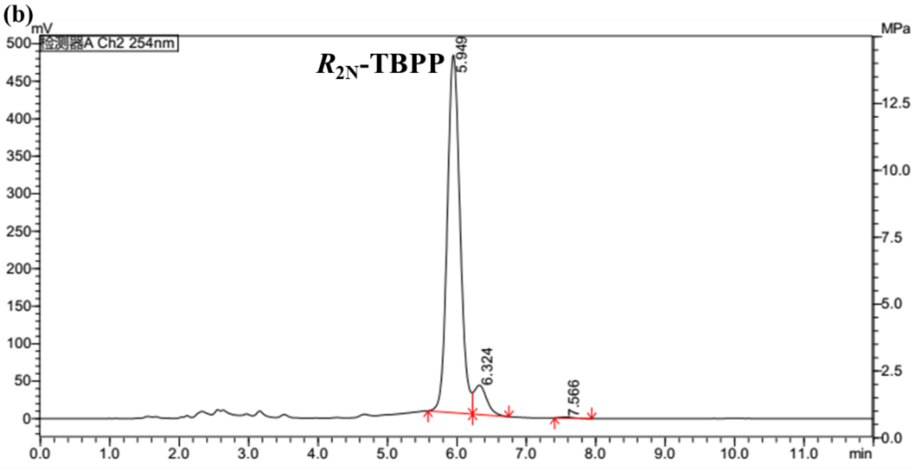


| Peak# | Ret. Time | Area | Area% | T.Plate# | Tailing F. | Resolution |
|---|---|---|---|---|---|---|
| 1 | 5.949 | 6120274 | 92.261 | 4906 | -- | -- |
| 2 | 6.324 | 496936 | 7.491 | 2571 | -- | 0.895 |
| 3 | 7.566 | 16471 | 0.248 | 7663 | 1.185 | 2.941 |

(c)

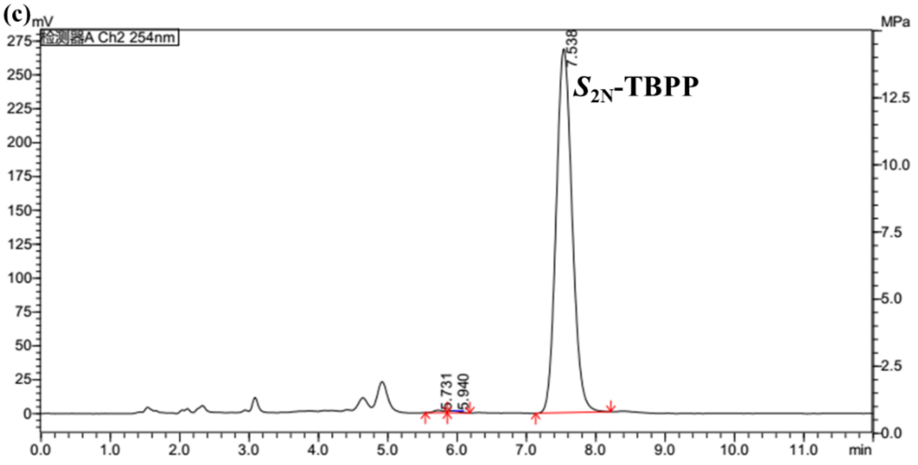


| Peak# | Ret. Time | Area | Area% | T.Plate# | Tailing F. | Resolution |
|---|---|---|---|---|---|---|
| 1 | 5.731 | 19446 | 0.448 | 4450 | -- | -- |
| 2 | 5.940 | 15549 | 0.358 | 2946 | -- | 0.537 |
| 3 | 7.538 | 4307267 | 99.194 | 5003 | 1.116 | 3.697 |

**Figure S5**. Resolution of the enantiomers [$R_{2N}$-**TBPP** and $S_{2N}$-**TBPP**] of *rac*-**TBPP** by a preparative chiral HPLC (a) *rac*-**TBPP**, (b) $R_{2N}$-**TBPP**, (c) $S_{2N}$-**TBPP**.

**4. Fabrication of the cogels**

**DGG** (20.0 mg, 48.0 mmol) was added to a capped test tube with **TBPP** ($3 \times 10^{-3}$ M for 1:16 and $4.8 \times 10^{-4}$ M for 1:80) chloroform solution (1.0 mL), the mixture was heated until the solid was dissolved completely. The solution was subsequently cooled down to room temperature. After 10 min, the gel formed and determined by the absence of flow of the solvent when the tube was inverted [S4].

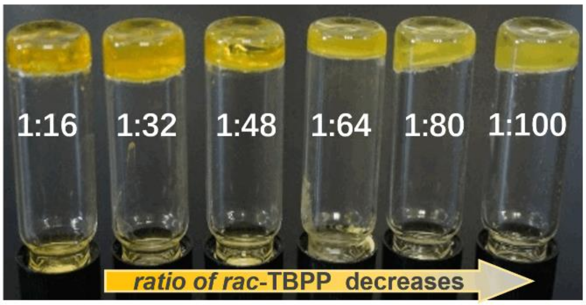


**Figure S6**. Cogels at different stoichiometric ratios of *rac*-**TBPP/DGG.**

**5. FT-IR absorption spectra of *rac*-TBPP/DGG cogels**

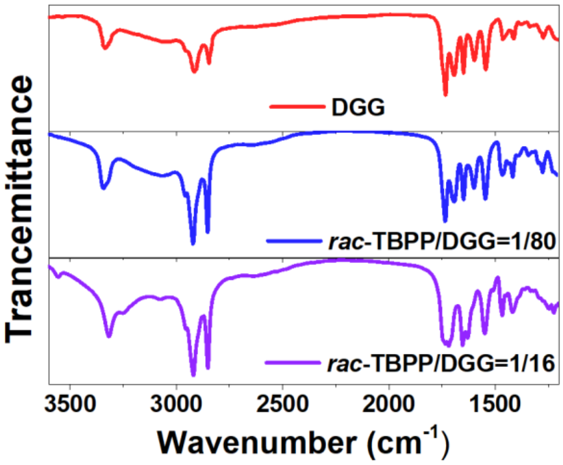


**Figure S7**. FTIR absorption spectra of *rac*-**TBPP/DGG** cogels at molar ratios of 1:80 and 1:16.

**6. Reference**

S1. Hu, X.; He, Y.; Wang, Z.; Yan, J. *Polymer* **2018**, *153*, 173-182. doi: 10.1016/j.polymer.2018.08.013

S2. Yuan, C.; Zhang, Y.; Xi, H.; Tao, X. *RSC Adv.* **2017**, *7*, 55577-55581. doi:10.1039/c7ra11228a

S3. Bachl, J.; Mayr, J.; Sayago, F. J.; Cativiela, C.; Díaz Díaz, D. *Chem. Commun.* **2015**, *51*, 5294-5297. doi:10.1039/C4CC08593K

S4. Li, P.; Lue, B.; Han, D.; Duan, P.; Liu, M.; Yin, M. *Chem. Commun.* **2019**, *55*, 2194-2197. doi:10.1039/c8cc08924h
